# Disseminated Intracranial and Spinal Neurenteric Cysts: A Case Report and Literature Review

**DOI:** 10.1155/2024/9673413

**Published:** 2024-10-25

**Authors:** Jan Kuschick Feher, Luis A. Marin-Castañeda, Fernando S. Juárez-Tovar, Roger Carrillo-Mezo, Gabriela Rosas, Martha Lilia Tena-Suck, Agnès Fleury

**Affiliations:** ^1^Peripheral Unit for the Study of Neuroinflammation, Instituto de Investigaciones Biomédicas, Universidad Nacional Autónoma de México, Mexico City, Mexico; ^2^Neurophysiology Department, Instituto Nacional de Neurología y Neurocirugía “Manuel Velasco Suárez”, Mexico City, Mexico; ^3^Neurocysticercosis Clinic, Instituto Nacional de Neurología y Neurocirugía “Manuel Velasco Suárez”, Mexico City, Mexico; ^4^Neuroradiology Department, Instituto Nacional de Neurología y Neurocirugía “Manuel Velasco Suárez”, Mexico City, Mexico; ^5^Faculty of Medicine, Universidad Autónoma del Estado de Morelos, Cuernavaca, Morelos, Mexico; ^6^Neuropathology Department, Instituto Nacional de Neurología y Neurocirugía “Manuel Velasco Suárez”, Mexico City, Mexico

**Keywords:** case report, intracranial cyst, neurenteric cyst, neurocysticercosis, spinal cyst

## Abstract

Neurenteric cysts (NECs) are rare congenital, benign lesions of the central nervous system (CNS), predominantly located within the spinal cord. However, they may also occur less frequently within the brainstem, fourth ventricle, or cerebellopontine angle (CPA). Originating from anomalous interactions between embryonic layers, NECs are recognized for their potential to compress adjacent structures. We report a unique case of disseminated NECs exhibiting few to absent symptoms, which represents an unusual presentation of this disease, with only six similar reports in the literature. A 22-year-old female presented to our institute with chronic headaches resistant to nonsteroidal anti-inflammatory drugs (NSAIDs). Initially treated for intracranial hypertension (ICH) secondary to a cyst in the quadrigeminal cistern at the age of 17 via neuroendoscopic surgery and subsequent ventriculoperitoneal shunting, she experienced transient relief. However, follow-up at 22 years of age revealed multiple cysts in the basal and spinal cisterns, with MRI findings suggestive of neurocysticercosis. Despite treatment with albendazole and corticosteroids, subsequent MRIs showed no change in the size or number of the cysts. Six years later, symptoms had worsened, previously identified cysts had grown, and the detection of new cysts prompted surgical intervention. Histopathological examination confirmed the presence of NECs. This case highlights the diagnostic challenges posed by NECs, especially in regions endemic for neurocysticercosis, where clinical and radiological findings may initially suggest this condition. It underscores the importance of considering NECs in the differential diagnosis of cystic lesions in the CNS, even in the absence of typical symptoms of spinal cord compression. The recurrence and spread of NECs post-treatment demand a comprehensive management approach, encompassing surgical intervention and close monitoring.

## 1. Introduction

Neurenteric cysts (NECs) also known as enterogenous or endodermal cysts, are rare, congenital benign lesions of the central nervous system (CNS) that predominantly manifest as singular spinal cystic masses [1]. These lesions, implicated in compression effects on the spinal cord, are less frequently observed in the brainstem, fourth ventricle, or cerebellopontine angle (CPA) [2, 3].

These cysts are derived from remnants of the primitive gut and notochord, reflecting their embryonic origin from misplaced endodermal cells during fetal development. This developmental anomaly leads to the formation of cysts that are lined by gastrointestinal or respiratory epithelium [3, 4]. The presentation of NECs can often mimic that of other cystic lesions in the CNS, such as arachnoid cysts or, as in the case presented, neurocysticercosis, particularly in regions where the latter is endemic. This overlap in radiological appearance makes accurate diagnosis challenging and often requiring definitive identification through surgical intervention and histopathological examination [2].

We report a unique case of multiple NECs affecting the cerebral and spinal compartments, showing clear growth, and causing few symptoms, representing an unusual presentation of this disease, complemented by a review of similar cases in the literature.

## 2. Case Description

**Figure 1:**
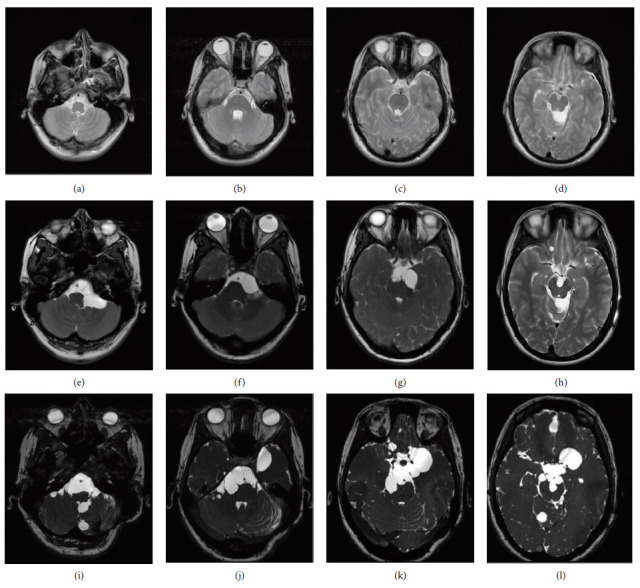
2010 T2-weighted (a)–(d), 2014 and 2020 FIESTA sequence ((e)–(h) and (i)–(l), respectively) magnetic resonance imaging (MRI). Slices at the level of the medulla oblongata (a), (e), (i), mid-pons (b), (f), (j), upper pons (c), (g), (k), and midbrain (d), (h), (l) showing the progressive increase in size of cysts.

A 22-year-old female patient presented at our institution with a chronic headache unresponsive to various NSAIDs. Despite a lack of clear triggers or associated neurological abnormalities, her medical history was significant due to previous neurosurgical interventions. The patient underwent neuroendoscopic surgery at 17 years of age (2009) to address intracranial hypertension (ICH) secondary to a unique cyst in the quadrigeminal cistern (not shown), which provided symptomatic relief. Despite this intervention, a ventriculoperitoneal shunt was required a year later due to recurrent ICH. Follow-up MRIs postsurgery (2010) showed no significant abnormalities, except for an enlargement of the quadrigeminal and left ambiens cisterns (Figures 1(a), 1(b), 1(c), and 1(d)). Following this intervention, the patient experienced no further symptoms, and subsequent neurological examinations were normal.

**Figure 2:**
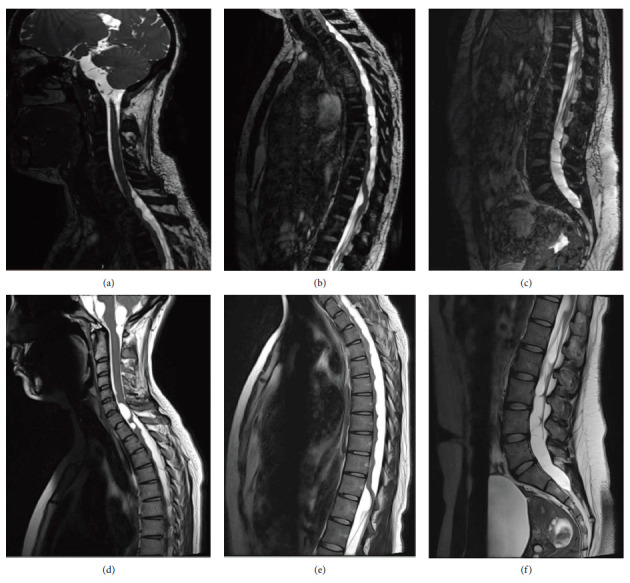
2014 CISS (a)–(c) and 2020 T2-weighted MRI (d)–(f). In (a) and (d), there are cysts in the posterior and anterior rachymedullar subarachnoid space; at the thoracic level in (b) and (e), there is a cystic conglomerate that compresses and displaces the spinal cord surface; in (c) and (f), multiple cysts displacing the nerve roots are observed.

At 22 years of age (2014), a follow-up MRI revealed multiple new cysts in the basal cisterns and surrounding the basilar artery causing compression of the pons, and additional cysts in the spinal subarachnoid space (Figures 1(e), 1(f), 1(g), and 1(h)). Despite the absence of spinal cord-related symptoms, an MRI of the spinal region revealed multiple cysts in the subarachnoid space within the spinal cavity (Figures 2(a), 2(b), and 2(c)). Lumbar cerebrospinal fluid (CSF) analysis yielded a cell count of 1/mm^3^, protein of 92 mg/dL, and glucose of 42 mg/dL. Based on radiological features and demographic characteristics a probable diagnosis of neurocysticercosis (NCC) was considered based on the criteria in use at that time [3]. A treatment regimen consisting of albendazole (30 mg/kg/day) and dexamethasone (0.1 mg/kg) was administered for 10 days without complications. Follow-up imaging with both CT and MRI scans revealed no change in the size or number of cysts.

A new lumbar CSF analysis yielded a cell count of 9/mm^3^, proteins of 75 mg/dL, and glucose of 39 mg/dL. Evaluation for specific NCC antigens (HP-10) and antibodies through ELISA yielded negative results in both serum and CSF. Despite these negative findings, the continued presence of radiologic evidence strongly indicative of NCC warranted the administration of a second course of treatment, consisting of albendazole (30 mg/kg/day for 15 days) and prednisone (1 mg/kg). Six months later, a follow-up MRI revealed no changes, and the patient remained asymptomatic.

**Figure 3:**
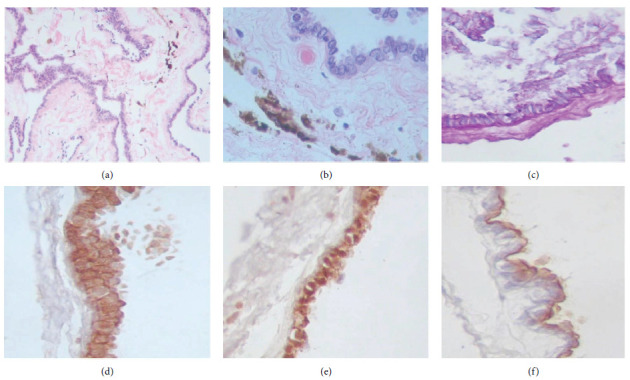
(a) Histological examination reveals a cystic lesion encased by columnar epithelium, which exhibits characteristics of pseudostratification. Within the stromal compartment, melanophages are indicative of pigmentary activity (Hematoxylin and Eosin stain, 200x magnification). (b) At higher magnification, the epithelial cells demonstrate a remarkable uniformity in appearance. The stroma, noted for its laxity, contains melanophages, underscoring the lesion's reactive stromal environment (Hematoxylin and Eosin stain, 400x magnification). (c) Utilizing Periodic Acid-Schiff (PAS) staining, a thickened basal membrane is observed, with the presence of PAS-positive material mirroring the composition of keratin, highlighting the lesion's complex extracellular matrix composition. Through immunohistochemical staining, the epithelium is shown to be constituted of cells positive for cytokeratin 6 (d) and cytokeratin 8 (e), a marker of epithelial lineage. (f) The employment of staining for epithelial membrane antigen (EMA) unveils a thin demarcating membrane encircling the epithelium's outer boundary, offering detailed visualization of the epithelial-stromal interface (400x magnification).

At 28 years of age (2020), the patient presented to the emergency department with severe holocranial headache, nausea, and tinnitus. Subsequent MRI imaging revealed an increase in the size of almost all cysts previously identified (Figures 1(i), 1(j), 1(k), and 1(l)). Notably, the only cyst that decreased in size was in the left pontocerebellar angle (Figure 1(j)). Surgical intervention was performed, during which some of the cysts were successfully fenestrated and removed. The macroscopic characteristics of the extracted cysts were suggestive of cysticerci; however, histopathological examination identified them as NECs (Figure 3). Immunohistopathological testing with specific antibodies targeting the cysticerci wall returned negative (Supporting Figure 1). A follow-up MRI conducted 6 months later demonstrated an increase in the number of cystic lesions within both the anterior and posterior subarachnoid spaces across all three segments of the spinal cord (Figures 2(d), 2(e), and 2(f)).

After the intervention, the patient continued a normal lifestyle and remains under continuous observation by the neurology and neurosurgery outpatient clinics. She experiences intermittent headaches, effectively managed with low doses of acetazolamide. Follow-up MRIs have shown a marginal increase in the number of cysts.

## 3. Discussion

We describe a rare case of a patient who developed multiple expanding cysts within the basal and spinal spaces, accompanied by hydrocephalus. Remarkably, the patient exhibited no motor or sensory deficits typically associated with medullary compression.

Originating from Mexico, a region where NCC is endemic, the patient's clinical manifestations and radiological signs initially suggested NCC, especially given the known link between cerebral NCC and asymptomatic spinal cysts [4]. However, the absence of a therapeutic response to albendazole, CSF characteristics that did not support this diagnosis (in cases of extraparenchymal NCC, the CSF exhibits high cellularity and protein contents and low glucose concentration), and negative antigen/antibody assays prompted further investigation. A conclusive histopathological examination revealed NECs as the final diagnosis.

NCC remains an endemic disease in several Latin American countries, where its incidence has generally shown a decreasing trend thanks to improved sanitary conditions in rural areas [5]. However, due to the persistent transmission of the infection in certain regions and the long delay between infection and symptoms (in cases of subarachnoid NCC, this delay can be up to 20 years), these cases continue to be diagnosed with some regularity in our country [6].

NECs are distinguished by their lining of mucin-secreting and/or ciliated, cuboidal to columnar epithelium, reminiscent of the respiratory and intestinal tract. These cysts have been identified by various names, e.g., enterogenous cyst, gastrocytoma, respiratory cyst, bronchogenic cyst, among others. Nevertheless, “neurenteric cyst” has emerged as the preferred term [7]. Despite their rarity in the CNS, where they comprise only 0.01% of tumors, NECs predominantly affect the spinal cord, often linked with dysraphic syndromes. Intracranial manifestations are exceptionally uncommon, with a predilection for the posterior fossa [7, 8].

Since their radiological features are not definitive, pathological analysis is crucial to distinguish NECs from other types of cysts. Typically, Hematoxylin-eosin staining reveals a lining of cuboidal or simple columnar epithelium, which may or may not have cilia, and often contains numerous goblet cells that secrete substances like mucin, which is believed to be the primary factor driving cyst growth [9, 10]. Furthermore, these cysts usually test immunopositive for markers such as cytokeratin, epithelial membrane antigen (EMA), and carcinoembryonic antigen (CEA), while showing immunonegativity for neuron-specific enolase (NSE), synaptophysin, glial fibrillary acidic protein (GFAP), and/or S-100 [10, 11]. This immunohistochemical profiling is essential for confirming the diagnosis. Notably, our patient exhibited positivity for EMA and cytokeratin (Figures 3(d), 3(e), and 3(f)), and negativity for GFAP (not shown), thereby confirming the diagnosis.

The presence of multiple NECs in both the intracranial and spinal regions of our patient is notably unusual. Upon conducting a literature review, we identified only six reported cases with extensive dissemination similar to ours [12–17] (Table 1). In some instances, cysts were identified along the spinal axis at initial diagnosis, while in others, widespread dissemination was observed years later. Remarkably, despite the extensive dissemination observed in our case, the patient exhibited minimal to no symptoms, a finding that appears to be unique.

NECs have a known tendency to regrow or spread, a phenomenon attributed to incomplete resection. To date, the patterns of recurrence are not fully understood, with only reported cases of intracranial dissemination following incomplete resection [13, 16]. This pattern of recurrence was also observed in our patient, who experienced an increase in the number of cysts after fenestration. Although total resection is the recommended approach for symptomatic patients, achieving total removal can be challenging due to the cysts' locations [1], as demonstrated in the presented case where complete resection was not feasible due to the extensive distribution of the cysts.

Our results highlight the need for additional research on this subject due to the challenges often encountered in diagnosis and the demand for new treatment approaches, especially in cases where surgery is not an option.

## Figures and Tables

**Table 1:** Characteristics of cases with multiple NECs.

Author and year	Age	Sex	Clinical presentation	Cyst location	Pathological findings		Management	Recurrence
					Histochemical	Immunohistochemical		
Perry et al. 1999 [13]	63	F	Ataxia, paraplegia, tremor, nausea	Posterior fossa and full length of spine	Simple cuboidal epithelium with papillary infoldings	+CK 7 +CK CAM 5.2 +CEA +EMA −CK 20 −GFAP −S-100	Resection	Yes
Oyama et al. 2004 [14]	46	F	Headache, vomiting, sixth nerve palsy, spasticity, dysesthesia	Bilateral cerebellar hemispheres, C2–C4	Single or multiple layers of columnar epithelial cells with secretory granules	+CK +EMA +CEA −GFAP −S-100	Gross total resection	Yes
Kimura et al. 2006 [15]	44	M	Gait disturbance, numbness, dysphagia, dysarthria, nystagmus	Bulbopontine, T4-5, T7-8, T12	Cuboidal or columnar epithelium, with intermingled goblet and ciliated cells	—	Subtotal resection	Yes
Yasuda et al. 2008 [12]	46	F	Nausea, dizziness, weakness	Subarachnoid space of the whole spinal canal	Cuboidal or columnar ciliated epithelial cells	+CK +CEA −GFAP	Cyst fenestration	No
Zarineh et al. 2009 [17]	20	F	Headache, dizziness	CPA, cerebellum	Nonciliated mucin-producing columnar epithelium with goblet cells	+CK 5/6/7 +Synaptophysin +CEA +EMA +BerEP4 −CK20 −TTF-1 −GFAP	Resection	Yes
Nosov et al. 2022 [16]	13	F	Headache, tinnitus, dizziness	Pineal, cerebellum, CPA, quadrigeminal cistern	Pseudostratified ciliated and partly cuboidal cells	+AE1/AE3 +EMA +CK 7 −GFAP −S-100 −CK20	Cyst fenestration	Yes
Feher et al. 2024 (present case)	22	F	Headache, nausea, tinnitus	Basal cistern and full length of spine	Columnar epithelium, melanophages in stromal compartment	+CK 6/8 +EMA −GFAP	Cyst fenestration	Yes

*Note:* +: immunopositive; −: immunonegative.

Abbreviations: CEA: carcinoembryonic antigen; CK: cytokeratin; CPA: cerebellopontine angle; EMA: epithelial membrane antigen; GFAP: glial fibrillary acid protein.

## Data Availability

Data sharing not applicable to this article as no datasets were generated or analyzed during the current study.

## Supporting Information

Supporting Information Supplementary Figure 1. Macroscopic appearance and immunohistochemical staining of cysts (anti-*T solium*). The first row displays a representative specimen showcasing characteristics of neurocysticercosis, while the second row illustrates findings from our patient.
