# Supplementary figures and images for: Disseminated Intracranial and Spinal Neurenteric Cysts: A Case Report and Literature Review
Source: Case Rep Neurol Med. 2024 Oct 25;2024:9673413. doi: 10.1155/2024/9673413 (PMC11530287; doi:10.1155/2024/9673413)

## Slide 1
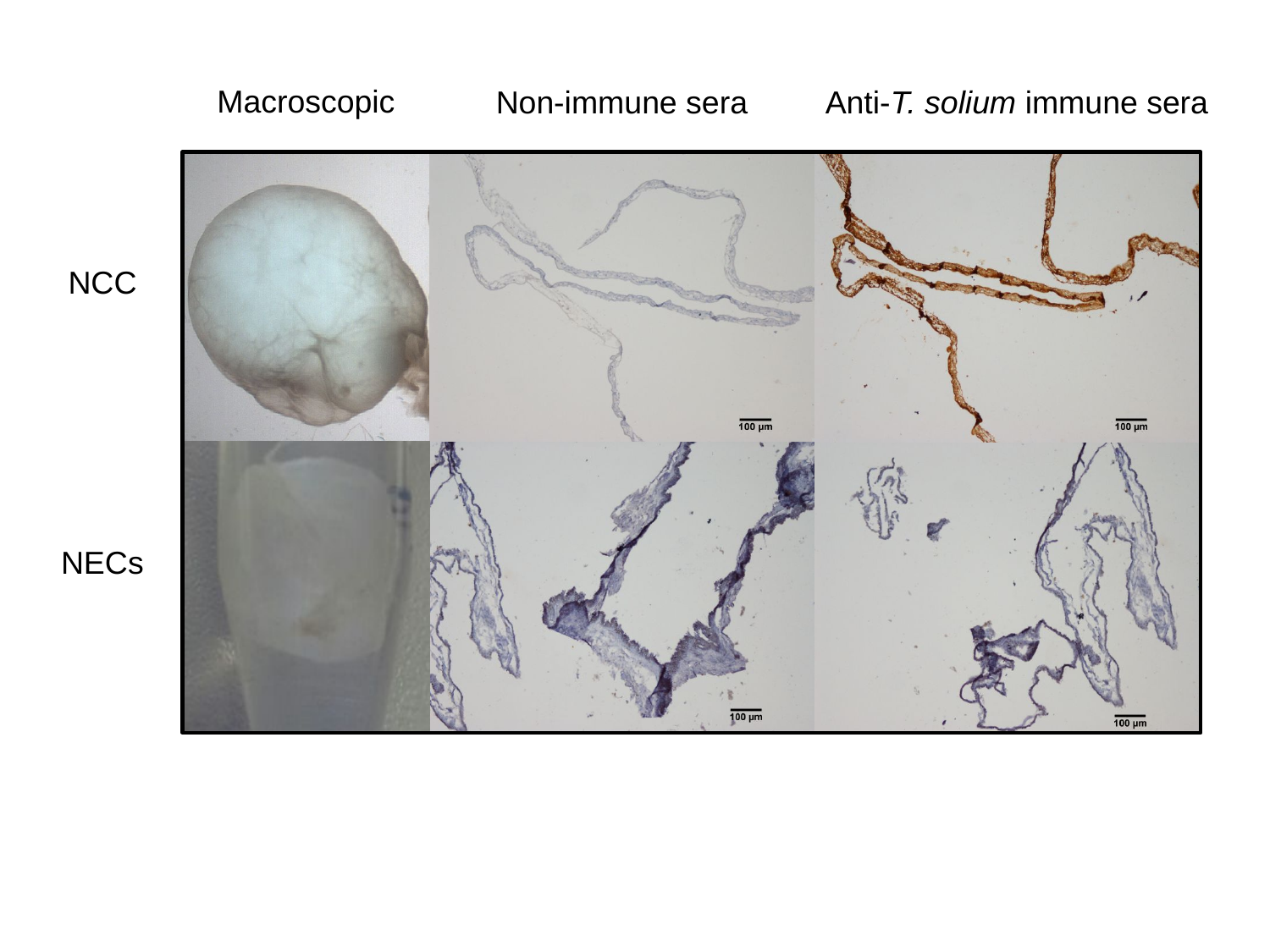

Macroscopic
Non-immune sera
Anti-T. solium immune sera
NCC
NECs

Supplement: Supporting Information — Supplementary Figure 1. Macroscopic appearance and immunohistochemical staining of cysts (anti-T solium). The first row displays a representative specimen showcasing characteristics of neurocysticercosis, while the second row illustrates findings from our patient. [file 9673413.f1.pptx]
